# Polymorphism in *heme oxygenase-1* (*HO-1*) promoter is related to the risk of oral squamous cell carcinoma occurring on male areca chewers

**DOI:** 10.1038/sj.bjc.6602186

**Published:** 2004-09-14

**Authors:** K-W Chang, T-C Lee, W-I Yeh, M-Y Chung, C-J Liu, L-Y Chi, S-C Lin

**Affiliations:** 1 School of Dentistry, National Yang-Ming University, Sec. 2, No. 155, Li-Nong St, Peitou, Taipei 112, Taiwan; 2 School of Life Sciences, National Yang-Ming University, Sec. 2, No. 155, Li-Nong St, Peitou, Taipei 112, Taiwan; 3 Department of Medical Education and Research, Veterans General Hospital, Taipei, Taiwan; 4 Oral & Maxillofacial Surgery, Taipei Mackay Memorial Hospital, Taiwan

**Keywords:** gene polymorphism, *HO-1*, mouth, neoplasm, oral submucous fibrosis

## Abstract

Areca (betel) chewing is associated with the high incidence of oral squamous cell carcinoma (OSCC) and oral submucous fibrosis (OSF) in Asians. *Heme oxygenase-1* (*HO-1*), encoding an oxidative response protein, plays protective roles in cells. A (GT)_*n*_ microsatellite repeat in *HO-1* promoter shows polymorphisms and modulates the level of gene transcription. We examined allelotypic frequencies of (GT)_*n*_ repeats in 83 controls, 147 OSCC and 71 OSF. All subjects were male areca chewers. Logistic regression was used to adjust the age confounding for odds ratio (OR). (GT)_*n*_ repeat polymorphism was classified into short (S), medium (M) and long (L) alleles. The adjusted OR in OSCC subjects carrying L allelotype relative to S allelotype was 1.75. Buccal squamous cell carcinoma (BSCC) is the most common OSCC subset in areca chewers. L allelotype implied the risk of BSCC with adjusted OR of 2.05, whereas M allelotype appeared protective for non-BSCC with adjusted OR of 0.49. Our findings indicated that longer (GT)_*n*_ repeat allele in *HO-1* promoter is associated with the risks of areca-related OSCC, while the shorter (GT)_*n*_ repeat allele may have protective effects for OSCC.

Heme oxygenase (HO) is a rate-limiting enzyme that degrades heme to produce biliverdin, CO and free iron. It was also named as heat shock protein 32 (Maines, 1988). Three HO isoforms have been identified in human. *Heme oxygenase-1* is an inducible isoform of HO in response to stresses, including heat shock, UV irradiation, hydrogen peroxide, heavy metals, glutathione deletion, hypoxia and NO (Maines, 1988; Shibahara, 1988; Keyse and Tyrrell, 1989; Oguro *et al*, 1996; Doi *et al*, 1999; Motterlini *et al*, 2000). The induction of *HO-1* represents a cytoprotective defence mechanism against oxidative insults. Like other heat shock proteins, high expression of *HO-1* was found in malignant tumours (Maines and Abrahamsson, 1996; Goodman *et al*, 1997). Administration of the HO inhibitor suppressed the growth of tumour cells, which suggests a vital role of *HO-1* in tumour growth (Doi *et al*, 1999; Fang *et al*, 2003). In addition, HO inhibitor also induced the apoptosis of tumour cells, suggesting the roles of *HO-1* in maintaining cancer cell survival (Fang *et al*, 2003). Reports have addressed that *HO-1* plays important roles in angiogenesis of prostate cancers (Sunamura *et al*, 2003).

Oral squamous cell carcinoma (OSCC) is the third most common malignancy in developing countries and the sixth worldwide. It accounts for up to 50% of malignant tumours in some South Asia countries due to the popularity of areca (betel)-chewing habit. Around 200–600 million Asians chew areca (Liu *et al*, 1996; Jeng *et al*, 2001; Lin *et al*, 2002; Lo *et al*, 2003; Sharma, 2003). OSCC is also a prevalent disease in Taiwan as the fourth leading malignancy in male population (Lin *et al*, 2000, 2002; Jeng *et al*, 2001; Kao *et al*, 2002; Lo *et al*, 2003; Wong *et al*, 2003). Areca was recently approved a carcinogen that produces oxidative stress and genotoxicity (Liu *et al*, 1996; Jeng *et al*, 2001; Bagchi *et al*, 2002; Sharma, 2003). Since buccal mucosa is the primary site for the insults of areca, buccal squamous cell carcinoma (BSCC) is the most common subset of OSCC accounting for more than 60% of OSCC in South Asians (Lin *et al*, 2000; Kao *et al*, 2002; Lo *et al*, 2003; Wong *et al*, 2003). It was found that a subset of OSCC, which occurred on tongue with higher *HO-1* expression, contained significantly more differentiated samples and cases without lymph node involvement, which implicated that *HO-1* expression could be disadvantageous for OSCC progression (Yanagawa *et al*, 2004).

Areca chewing is also exclusively associated with the occurrence of oral submucous fibrosis (OSF), which is a precancerous condition exhibiting disturbances in homeostasis of fibrous tissue and altered epithelial components (Chiu *et al*, 2001, 2002; Yang *et al*, 2001; Ko *et al*, 2003; Liu *et al*, 2004; Shin *et al*, 2004). Oral submucous fibrosis was originally called idiopathic scleroderma of the mouth. This disease is characterised by mucosal rigidity due to the fibro-elastic transformation of the juxta-epithelial layer. A subepithelial inflammatory reaction and epithelial atrophy is frequently accompanied with the fibrous changes of the lamina propria (Shin *et al*, 2004). It is interesting that OSF seems to be a public health issue in many parts of the world, including the UK and South Africa, due to the spreading of areca chewing (Canniff *et al*, 1986; Yang *et al*, 2001; Shin *et al*, 2004). However, the genetic susceptibility or disease nature of OSF is still largely undefined.

(GT)_*n*_ repeats were identified in the proximal region of *HO-1* promoter. Reports indicated that these microsatellites are highly polymorphic, and longer (GT)_*n*_ repeat exhibits lower *HO-1* transcriptional activity (Yamada *et al*, 2000; Chen *et al*, 2002, 2004; Kaneda *et al*, 2002). Subjects carrying longer (GT)_*n*_ repeats are associated with the higher susceptibility of cardiovascular or chronic obstructive pulmonary diseases (Yamada *et al*, 2000; Chen *et al*, 2002, 2004; Kaneda *et al*, 2002). Oxidative stress is associated with the pathogenesis of cancers (Xie and Huang, 2003). However, the association between *HO-1* promoter polymorphism and cancer risk has not been established. Although *HO-1* expression might be related to the biological behaviour of OSCC (Yanagawa *et al*, 2004), the involvement of functional *HO-1* polymorphisms in the risk of oral diseases has not been defined. We hypothesised that higher (GT)_*n*_ repeats may be associated with the risk of OSCC and OSF. We showed that (GT)_*n*_ repeats polymorphism in *HO-1* was a risk factor for OSCC. In addition, polymorphic profile differences seemed to differentially predict BSCC and non-BSCC subsets.

## MATERIALS AND METHODS

### Samples

A total of 147 primary OSCC without previous treatment and 71 OSF cases were obtained from the Oral and Maxillofacial Surgery Department of the Taipei Mackay Memorial Hospital. In all, 83 control subjects were selected from people who came for physical checkup, and had no neoplastic minor oral operations or maxillofacial trauma. Those with autoimmune disorders, blood diseases and previous malignancies were excluded from the control group. The site, stage and TNM classification of OSCC subjects are described in Table 1

**Table 1:** Clinical parameters of OSCC subjects

*Site*	
Buccal mucosa	90
Tongue	35
Gingiva	14
Palate	5
Floor of mouth	3
	
*Stage and TNM*^a^	
Stage I	15
Stage II	35
Stage III	20
T1N1M0	2
T2N1M0	5
T3N0M0	11
T3N1M0	2
Stage IV	77
T2N2M0	3
T3N2M0	3
T4N0M0	32
T4N1M0	24
T4N2M0	8
T4N3M0	7

a According to UICC classification system.

. This study was approved by an ethics reviewing committee. Blood was drawn from the subjects. A leukocyte cell pellet was obtained from the buffy coat by centrifugating the whole blood. DNA was isolated by Blood Mini Kit (Qiagen, Valencia, CA, USA).

### *Heme oxygenase-1* genotyping

(GT)_*n*_ repeat polymorphism in the *HO-1* promoter was determined by PCR-based genotyping. The primers used to generate *HO-1* amplicons of 98–142 bp were sense: 5′-AGAGCCTGCAGCTTCTCAGA-3′ and antisense: 5′-ACAAAGTCTGGCCATAGGAC-3′ (Kaneda *et al*, 2002). The 5′ site of the sense primer was labelled with FAM fluorescence dye. The amplification reaction mixture (15 *μ*l) contained 20 ng genomic DNA, 0.2 mM of each dNTP, 0.5 *μ*M of each primer, 0.5 U Prozyme DNA polymerase (Protech Enterprise, Taipei, Taiwan) and 1 × PCR buffer. The PCR reaction was carried out in three steps: firstly, 2 min at 94°C; then, 30 cycles of 30 s at 94°C, 30 s at 56°C and 30 s at 72°C; lastly, 5 min at 72°C. The amplicons were denatured for 5 min at 100°C, and mixed with formamide-containing stop buffer, and then subjected to electrophoresis on 4% polyacrylamide gel containing 8 M urea in an ABI Prism 377-18 DNA sequencer (Applied Biosystem, Foster City, CA, USA). The fluorescence was detected automatically by Genescan 672 software (Applied Biosystem). At least two independent experiments were performed on each sample to assure the reliability of the analyses.

### DNA sequencing

Selected amplicons with various allelic sizes were cloned into pGEM-T vector (Promega, Madison, MI, USA). Five clones from each allele were sequenced using a 377-18 DNA sequencer (Applied Biosystem) and vector primers were used to confirm the number of GT repeats revealed by genotyping.

### Statistical analysis

Associations between the *HO-1* polymorphisms and risk of disease genesis were estimated by odds ratio (OR) and associated 95% confidence interval (CI), which were calculated by logistic regression models using SPSS version 8.0 (SPSS Inc., Chicago, IL, USA). Differences between the variants were considered significant when *P*<0.05.

## RESULTS

All subjects were male areca chewers. The ages (mean±s.d.) of OSCC, OSF and control subjects were 51.3±9.8, 39.0±10.8 and 47.1±10.0, respectively. In all, 61% (90 cases) of the OSCC subjects were BSCC, 37% (54 cases) of the OSCC patients presented with lymph node metastasis (LNM) and 63% (97 cases) of patients had late stage lesions (Table 1).

**Figure 1:**
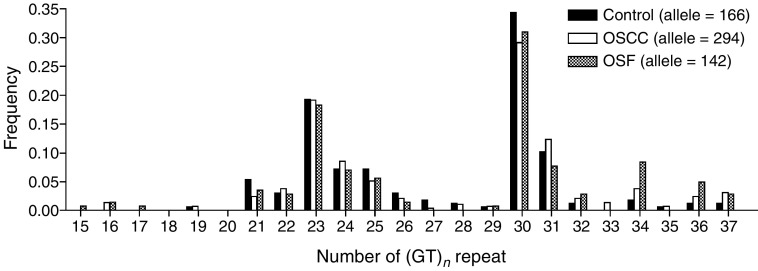
Allelotypic distribution of *HO-1* (GT)_*n*_ repeat polymorphisms in control, OSCC and OSF subjects.

The genotyping of (GT)_*n*_ microsatellite polymorphism in *HO-1* promoter region was carried out by Genescan system. It distinguished (GT)_*n*_ repeats on the basis of differential mobility of amplicons with different sizes. The repeat numbers were derived from the amplicon size and the sequencing reading in cloned alleles. The allelotypic distribution of *HO-1* polymorphism of control, OSCC and OSF is shown in Figure 1

. In controls, the GT repeat numbers ranged from 19 to 37, and the most common alleles were (GT)_23_ and (GT)_30_, which were consistent with previous Taiwanese studies (Chen *et al*, 2002, 2004). The alleles were classified into three subgroups: the shorter component (⩽25 repeats) was designated as class ‘S’, the medium component (26–30 repeats) was designated as class ‘M’ and the longer component (⩾31 repeats) was designated as class ‘L’. Table 2

**Table 2:** *HO-1* genotype in subjects

**Genotype**	**Control (*n*=83)**	**OSCC (*n*=147)**	**OSF (*n*=71)**	**BSCC (*n*=90)**	**Non-BSCC (*n*=57)**
SS	17	29	7	14	15
SM	29	30	23	17	14
SL	11	34	20	19	15
MM	13	20	8	16	4
ML	12	27	8	20	6
LL	1	7	5	4	3

describes the genotypes from Genescan analysis. Table 3

**Table 3:** Association between *HO-1* allelotype and risks of OSCC and OSF subjects

	**Control**	**OSCC**				**OSF**			
	***n***	***n***	***P***	**OR**	**95% CI**	***n***	***P***	**OR**	**95% CI**
S	74	122		1.00		57		1.00	
M	67	97	0.581	0.88	0.57–1.34	47	0.796	0.91	0.55–1.51
L	25	75	0.030	1.81	1.06–3.10	38	0.035	1.95	1.06–3.59
L^a^			0.045	1.75	1.01–3.03		0.160	1.61	0.83–3.13

a Adjusted for age.

describes that the frequency of L allelotype was significantly increased in OSCC subjects in relation to control subjects, with an age-adjusted OR of 1.75. The frequency of L allelotype was also significantly increased in OSF subjects relative to control subjects before adjusting. However, after adjusting for age, OR remarkably declined to an insignificant level (Table 3).

**Figure 2:**
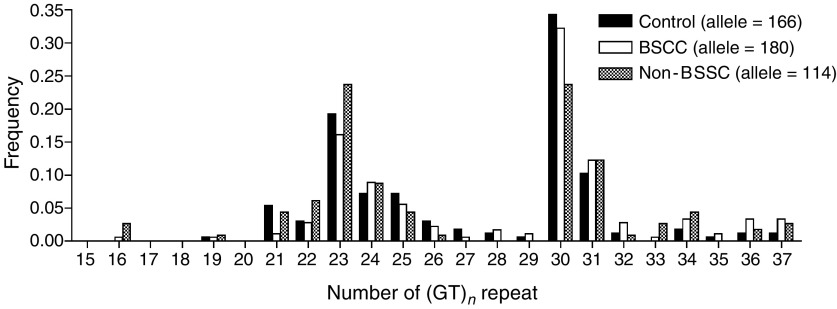
Allelotypic distribution of *HO-1* (GT)_*n*_ repeat polymorphisms in control, BSCC and non-BSCC subjects.

Further analysis on different clinical parameters, including site, LNM and stage, was performed to elucidate the relationship between *HO-1* polymorphism and OSCC. The allelotypic distributions of *HO-1* polymorphism in control, BSCC and non-BSCC subjects are shown in Figure 2

. A significantly higher difference in frequencies of L allelotype was noted in BSCC subjects compared with control subjects (Table 4

**Table 4:** Association between *HO-1* allelotype and risks of BSCC and non-BSCC subjects

	**Control**	**BSCC**				**Non-BSCC**			
	***n***	***n***	***P***	**OR**	**95% CI**	***n***	***P***	**OR**	**95% CI**
S	74	64		1.00		59		1.00	
M	67	69	0.546	1.19	0.74–1.91	28	0.013	0.49	0.27–0.85
M^a^							0.011	0.49	0.28–0.85
L	25	47	0.011	2.14	1.19–3.87	27	0.413	1.36	0.71–2.58
L^a^			0.019	2.05	1.12–3.73				

a Adjusted for age.

). Interestingly, the frequency of M allelotype in non-BSCC subjects was significantly reduced in relation to control subjects (Table 4). Analyses revealed no statistically significant difference in the *HO-1* polymorphism in OSCC subjects that exhibited different LNM and clinical stage (detailed analysis not shown).

## DISCUSSION

In this case–control study, we have identified a significant association between GT repeat length in *HO-1* promoter and the risks of OSCC occurring in male areca chewers, particular for the BSCC subset. Increases in risk were observed for subjects with a GT repeat ⩾31, which suggested that shorter GT repeat alleles could have a protective effect on OSCC. The results also supported previous findings suggesting better protection against disease formations in subjects carrying shorter *HO-1* GT repeats (Yamada *et al*, 2000; Chen *et al*, 2002, 2004; Kaneda *et al*, 2002). Since *HO-1* is critical for converting heme to bilirubin, CO and iron, we have examined the correlation between *HO-1* polymorphisms and serum bilirubin to support the notion that subjects with longer GT repeat might have higher serum bilirubin. Although we observed a trend that subjects carrying L allelotype have a higher serum bilirubin level, the increase did not reach a statistical significance (detailed analysis not shown). It is plausible that the induction of *HO-1* only occurs in local tissue and may not necessarily stand out from measuring circulating bilirubin level, since serum bilirubin level is affected by multiple systemic factors including liver function. Male areca chewers with L allelotype have 1.75 or 2.05 times higher risk for OSCC or BSCC, respectively, compared to those carrying S allelotype. To our knowledge, this is the first study investigating the roles of *HO-1* promoter polymorphism on OSCC risks.

Oral submucous fibrosis is a unique disease characterised by the unbalance between synthesis and degradation of extracellular matrix (Chiu *et al*, 2002), and occurred exclusively in areca chewers (Chiu *et al*, 2001, 2002; Ko *et al*, 2003; Liu *et al*, 2004). It is considered as an inflammatory reaction in response to areca ingredients or physical irritation, while the covering epithelium may exhibit precancerous changes undertaking malignant transformation (Ko *et al*, 2003). The fact that areca disrupts cytokine production and molecules for organising the extracellular matrix seems to play important roles in OSF pathogenesis. Studies have demonstrated that the functional polymorphism of genes on immune reaction, such as *MICA* (Liu *et al*, 2004), *CTLA4* (Shin *et al*, 2004), collagen-related genes (Chiu *et al*, 2002) and cytokines (Chiu *et al*, 2001), were associated with the risk of OSF. The OSF patients usually came for medical helps for resolution of oral symptoms early in their lives. Thereby, the mean age of OSF subjects is around 10 years younger than control or OSCC subjects in our study cohort. In this study, the risk of L allelotype in *HO-1* promoter for OSF became not significant after adjusting for age. It was speculated that the protection driven by *HO-1* is not sufficient for counteracting the oxidative stress elicited by areca, which might cause the pathogenesis of OSF (Liu *et al*, 1996). Age-related confounding factors or genetic events contributive to OSF genesis deserve further dissection.

We identified that the longer GT repeat length was highly associated BSCC. In contrast, medium GT length was inversely associated with non-BSCC. The data suggest that *HO-1* polymorphism might have a profound effect on the risk of getting carcinomas at different oral locations. Buccal squamous cell carcinoma accounts for more than 60% of the total areca-associated OSCC, but it is extremely rare in the West (Lin *et al*, 2002, 2004; Lo *et al*, 2003). Previous studies from us have specified the great molecular discrepancies between BSCC and non-BSCC (Lin *et al*, 2000; Kao *et al*, 2002). Interestingly, the frequency of a functional genotype in *CCND1* and *MMP-1* was also contradictory between BSCC and non-BSCC (Wong *et al*, 2003; Lin *et al*, 2004). In the present study, we further proposed the distinctive *HO-1* promoter polymorphisms between BSCC and non-BSCC. Evidences accumulated might suggest that BSCC, which is quite prevalent in Asians, exhibits distinctive pathways for tumorigenesis.

*Heme oxygenase-1* expression has been reported to enhance growth against apoptosis and induce angiogenesis through increase in angiogenic factor and VEGF in endothelial cells and cancer cells (Deramaudt *et al*, 1998; Doi *et al*, 1999; Kushida *et al*, 2002; Malaguarnera *et al*, 2002, 2003; Fang *et al*, 2003; Sunamura *et al*, 2003). Such phenotypes are advantageous for tumour progression and survival. However, a recent paper, denoting the lower *HO-1* expression in head and neck carcinomas with LNM, has argued that the increase of *HO-1* expression as a cause or a consequence of carcinogenesis (Yanagawa *et al*, 2004). Preliminary evidences from us indicated that *HO-1* promoter polymorphism had no impact on tumour progression, reflecting by metastasis or advanced clinical stage. It might exclude the role of *HO-1* polymorphism as a risk marker of tumour dissemination, although such polymorphism could affect transcription. This could be partially interpreted by the multiple factors involving in the tumour progression, which mask the crucial role of *HO-1* genotype. Advanced tumours exhibited tremendous potentials to survive in a stress microenvironment, with hypoxia and free radical overproduction (Xie and Huang, 2003). Thereby, the extensive stress on advanced tumours may have secondary or selective effects on *HO-1* expression. Additional genotypic surveys using more samples and confounders, together with *HO-1* expression profile, are required to further insight the functional importance of *HO-1* in cancer progression.

Overall, our clues indicated that longer GT repeat allele in *HO-1* promoter is associated with the risks of areca-associated oral carcinogenesis. The findings also suggest that shorter GT repeat allele in *HO-1* promoter may have protective effects for OSCC in our study cohort.
